# Personalized Design of 3D-Printed Osteochondral Scaffold for Osteoarthritis Patients with Different Bone Conditions and Mechanical Evaluation

**DOI:** 10.3390/bioengineering12111226

**Published:** 2025-11-10

**Authors:** Jian Zhou, Leixin Liu, Peixuan Zhi, Yanan Dong, Ziyu Liu, Yubo Fan

**Affiliations:** 1 University of Health and Rehabilitation Sciences, Qingdao 266011, China; spine@aliyun.com; 2 Beijing Advanced Innovation Center for Biomedical Engineering, School of Engineering Medicine, Beihang University, Beijing 100191, China

**Keywords:** scaffold structure, 3D printing, knee joint bioreactor, mechanical simulation, osteoarthritis

## Abstract

As osteoarthritis is a common disease in elderly people and large cartilage defects can only be treated by joint replacement surgery, a scaffold is seen as a potential treatment that could help patients to delay or avoid surgery. An ideal scaffold should have similar properties to the surrounding tissues. Thus, for different levels of OA, patients with different bone properties should use different scaffold structures with different mechanical or biological properties. In this paper five structures (A–E) are designed for young OA patients or patients with good bone mechanical properties, middle-age OA patients with weak bone mechanical properties or patients with little osteoporosis, and elderly OA patients who have severer OA and osteoporosis who are not able to perform normal activities. And these five scaffold structures are 3D-printed by an EOS machine with Ti6Al4V powder and evaluated by experiments based on a biomechanical bioreactor simulating the human knee joint and simulation through ANSYS. Structure D with a solid thick beam in the middle has the highest loading force, which is 3707.835 N, and structure E, composed of the polyhedron with the highest specific surface area, has the lowest loading force, which is 1837.402 N. Structures A, B, and C are intended for young OA patients or patients with good bone mechanical properties. Structures D and E are designed for patients who need to avoid or delay joint replacement surgery.

## 1. Introduction

Osteoarthritis (OA) is a prevalent disease among the elderly, often requiring joint replacement surgery to treat large cartilage defects [1,2]. Osteochondral scaffolds have shown promising potential in the repair of cartilage and bone, providing a suitable mechanical and biological environment for regeneration [3,4,5,6,7,8]. These scaffolds must be integrated into surrounding tissues, such as cartilage, the subchondral bone plate, and trabecular bone, while ensuring cell viability, proliferation, differentiation, and other related processes [9,10,11,12,13].

The ideal scaffold should provide suitable biomimetic, mechanical, and biological environments, while maintaining similar morphological and functional features to osteochondral bone to optimize integration into neighboring tissues [4,14,15,16,17]. Furthermore, personalized scaffolds may be more suitable for different types of OA patient. For instance, young OA patients with high bone density and strength may require scaffolds with high porosity and strength due to their superior healing ability and higher activity levels. Conversely, patients suffering from both OA and osteoporosis may require scaffolds with high porosity and weaker strength, as their surrounding tissues cannot withstand high loadings and their activity levels are likely lower.

Therefore, the geometry and mechanical properties of scaffolds should be thoroughly investigated to meet the diverse requirements of different types of OA patients. These factors play crucial roles in tissue engineering, influencing cell absorption, distribution, proliferation, differentiation, and migration [18,19,20]. Typically, scaffolds are designed as parallelepipeds with square cross-section channels or composed of different filaments with varying orientations [21,22]. However, as cells consume nutrients and oxygen, a gradient density from the outside to the inside of the scaffold is created, enabling cell movement [23,24]. Given the time it takes for nutrients to move from the outside to the inside of a scaffold, a gradient structure or polyhedron spatial structure might be more suitable [25,26,27,28].

Due to the high cost and time-consuming nature of in vivo and in vitro tests for evaluating scaffolds, a novel bioreactor is needed to help researchers avoid extensive testing and provide a similar bioenvironment. Tissue engineering bioreactors can be broadly categorized into two types. The first is akin to a perfusion culture system, providing a suitable, biological, and dynamic environment to accelerate cell/tissue maturation [29]. Eghbali et al. developed a perfused bioreactor to investigate the effects of nutrient transport and culture parameters on scaffold cell growth, finding that scaffolds cultured under perfusion were 30% more effective than those cultured under static conditions [30]. Pisanti et al. found that a tubular perfusion system could enhance hMSC proliferation and differentiation and could also magnify the effects of the architecture of the scaffold [31]. However, a limitation of many perfusion bioreactors is that they only use one nutrient solution or mix several nutrient solutions together to enhance cell growth [32]. In a real bioenvironment, different tissues are not in the same fluid environment, and some fluids may inhibit the regeneration of other cells or tissues, such as synovial fluid preventing bone formation [33,34].

The second type of bioreactor is more like a bionic system, attempting to mimic internal biological environments in the human body that are difficult to observe or investigate [35]. Olivares et al. [36] used Melchels et al.’s [37] bioreactor experiment results to investigate a numerical model for simulating cell and scaffold interactions.

In this paper, we develop five different scaffold structures to fit different types of OA patient and a biomechanical bioreactor simulating a human knee joint. These five types of scaffolds are evaluated using a bioreactor in a biomechanical field. Additionally, finite element models for the five types of scaffolds tested in the bioreactor are developed to explore the structure design.

## 2. Materials and Methods

### 2.1. Design and Manufacturing Method for the Scaffold

**Figure 1:**
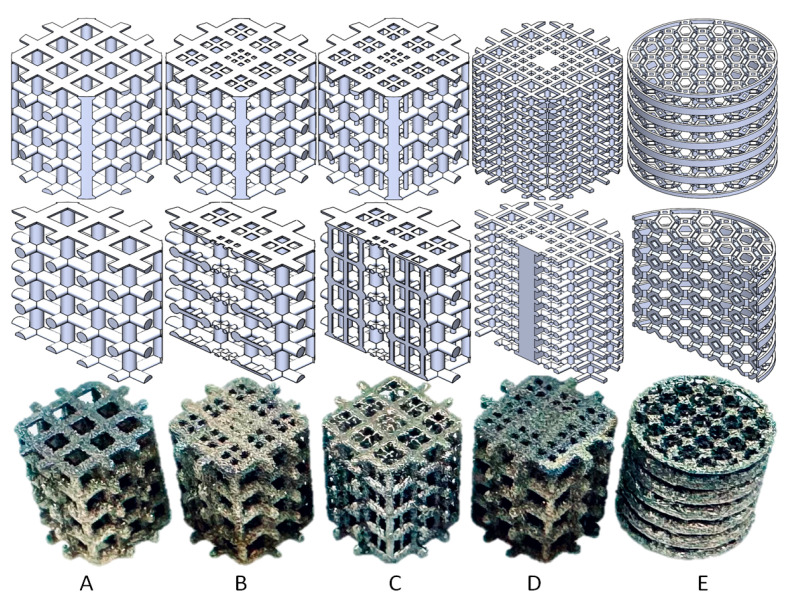
Five types of scaffold (**A**–**E**) structure for different stages of OA.

We designed five porous scaffolds with different crosslink structures or truncated octahedron (TO) structure. These were manufactured using an EOS M290 machine (EOS GmbH- Electro Optical Systems, Krailling, Germany) with EOS Titanium Ti64 (Ti6Al4V) powder through the direct metal laser sintering (DMLS) method. The scaffolds were treated with a 650 °C normalizing stress relief treatment and a 3 h annealing process as shown in Figure 1 [38]. The additive-manufactured (AM) items of the titanium alloy (Ti6Al4V) were based on the maximum principal stress criterion, and failure would occur if the maximum tensile stress exceeded 1100 Mpa.

The five scaffolds are categorized into three types. Structures A, B, and C are designed for young OA patients or OA patients with high bone strength. Structure D is intended for middle-aged OA patients or OA patients with lower bone strength and mild osteoporosis. Structure E is designed for elderly patients or OA patients with severe osteoporosis. The design index for structures A, B, C, and D includes 5 Mpa pressure, as patients with light or middle-level OA still want to perform suitable activities. However, structure E is designed to help OA patients delay or avoid joint replacement surgery, with a design index with 1 Mpa pressure, as these patients are only able to perform limited activities.

The outer envelope of these five types of scaffolds is a cylinder with a diameter of 6.6 mm and a height of 6 mm, and the bodies of the scaffolds are all center-symmetric structures. Structures A, B, and C increase in complexity, with A being one of the most common designs and C being specifically designed for young OA patients with slightly more serious symptoms [38,39].

Structure A is designed with 0.5 mm diameter columns that are vertically cross-linked, and the pore size on the top surface is 1 mm × 1 mm. Structures B and C are gradient structures with 0.5 mm diameter columns vertically cross-linked, and the pore size on the top surface ranges from 0.4 mm × 0.4 mm on the side to 0.2 mm × 0.2 mm in the middle. However, structure C has additional 0.2 mm diameter axial support cylinders vertically placed in the middle of the structure.

Structure D is designed with 0.25 mm diameter cross-linked columns, and the top surface pore size shows a gradient decreasing from the side to the middle, from 0.8 mm × 0.8 mm to 0.3 mm × 0.3 mm to none (0.8 mm × 0.8 mm solid cuboid).

Structure E is a 3D structure constructed by a truncated octahedron (unit cell with 1 mm height and 1 mm width) with 0.1 mm thick round rings. The design is made by extruding a cut of an octagonal prism (formed by square sides and two regular octagonal caps) with 14 faces (8 hexagons and 6 squares) and 36 sides [35,38].

### 2.2. Design of the Bioreactor

**Figure 2:**
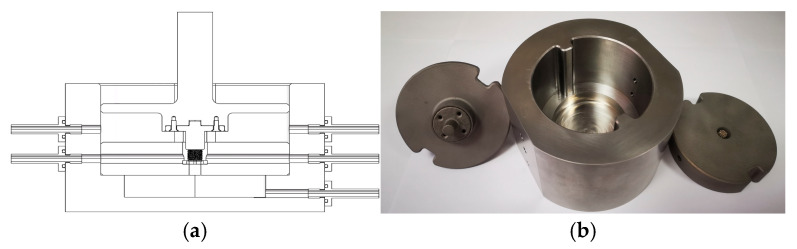
Human knee joint bioreactor design ((**a**) schematic diagram; (**b**) physical object).

To meet the requirements of providing a perfusion culture system for enhancing cell/tissue growth and mimicking the internal human body’s biological environment, we developed a bioreactor simulating a human knee joint (Figure 2). The bioreactor has five chambers located symmetrically on the side, all of which can perfuse fluid into the system and can also be closed. There is a plunger in the middle that can be coupled with a universal testing machine to provide loading.

### 2.3. Bioreactor Mechanical Test

**Figure 3:**
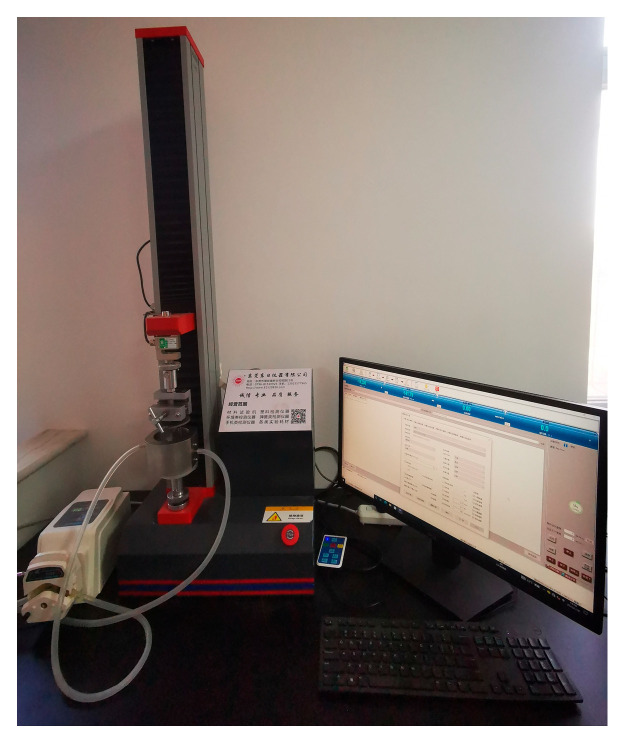
Scaffold evaluation using human knee joint bioreactor.

The five types of scaffolds are fixed in the holder with a 3.3 mm radius cylindrical hole as shown in Figure 3. Before starting the experiment, the bioreactor is filled with phosphate-buffered saline (PBS). A peristaltic pump is connected to two chambers, and the other chambers are closed. The loading speed of the plunger is set to 60 mm/min, and the preloading force is 0.1 N. The stroke of the plunger is 3.5 mm, and the precision of the sensor is 0.1% with a maximum range of 5000 N. During the loading test, the peristaltic pump is working to circulate the flow of the PBS in the bioreactor.

### 2.4. Scaffold Performance Simulation Model

**Figure 4:**
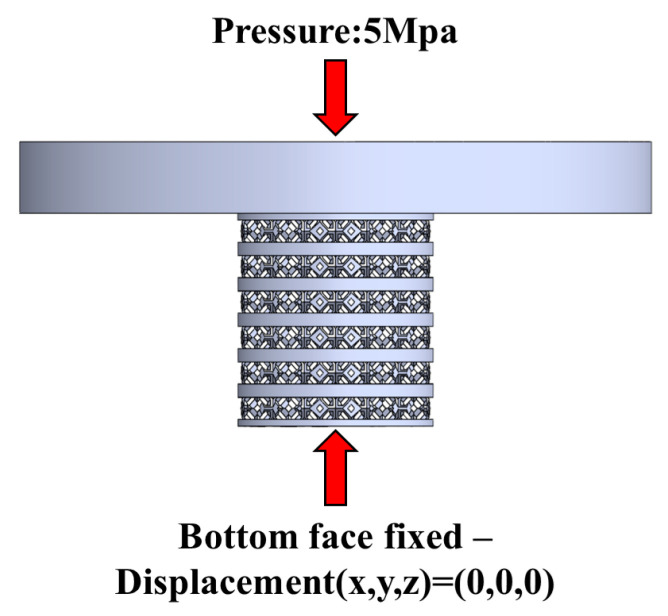
Boundary conditions of scaffold simulation.

To investigate the stress distribution of all types of scaffolds during loading, we simplified finite element (FE) models to a plate applying pressure to the scaffolds using ANSYS 2021 R1 version. The scaffold material property is set with a density of 4.41 × 103 kg/m^3^, a Young modulus of 6500 Mpa, and a Poisson ratio of 0.342 [40]. A 5 Mpa pressure is applied using a 20 mm diameter plate. The boundary condition diagram is shown in Figure 4, where the loading pressure for structures A–D is 5 Mpa, and for structure E, it is 1 Mpa. The bottom face is set as fixed, simulating the scaffold’s real situation in the bioreactor with displacement in all three directions.

## 3. Results

The design logic for these five different structures is based on three factors that need to be balanced: cell/tissue growth, stress shielding, and bone remodeling. Compared to the common scaffold design (structure A), structures B, C, and D incorporate a gradient structure for cell growth, considering that nutrient concentration is distributed in a gradient within the scaffold structure when implanted in human or animal bodies, with the lowest concentration occurring in the middle of the scaffold [24].

Unlike the other structures, structure E is designed for elderly OA patients with osteoporosis. This scaffold is intended to substitute the function of the osteochondral tissue with OA that is removed during surgery. As elderly patients’ cell activity is not as robust as that of younger patients, the structure is designed with high porosity and a large specific area to aid bone mesenchymal stem cell (BMSC) attachment [2,38].

All the structures are designed to avoid the stress-shielding effect. Patients should choose the scaffold with mechanical properties that are similar to their bone properties [40]. According to the bone remodeling theory, suitable bone micro-strain and stress enhance bone growth [41], so the edge of the scaffold should be designed to have sufficient surface area in contact with the surrounding tissues. However, as the specific mechanism of bone remodeling is still unclear, the structure could still be improved in the future.

### 3.1. Experimental Evaluation of the Scaffold Structure

**Figure 5:**
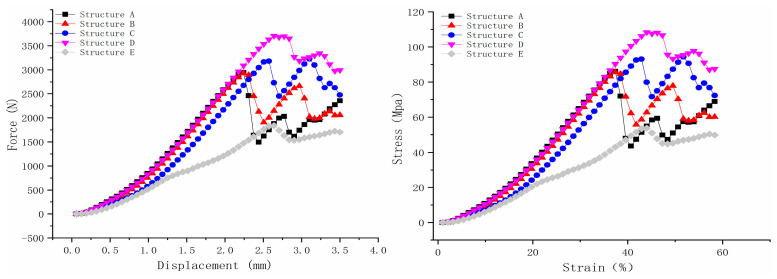
Scaffold bioreactor mechanical test for 5 structures of scaffolds (force–displacement; stress–strain).

The five scaffold structures were evaluated using a bioreactor filled with PBS, and the results are shown in Figure 5. According to the force–displacement curve, structure D exhibited the highest maximum loading force, which was 3707.835 N. Structures A and B demonstrated nearly the same maximum force, which were 2945.087 N and 2931.775 N, respectively. Structure C displayed two peaks, similar to structures A, B, and D, but the second peak’s maximum force was higher than the first peak. After the first peak point, some beams in the scaffold structure would fail. Therefore, the maximum force of structure C was selected as the first peak value, which was 3183.281N. Structure E showed the lowest loading force, which was 1837.402 N.

As for the stress–strain curve, structures A and B also showed nearly the same ultimate strength, which was 86.084 Mpa and 85.695 Mpa, respectively, as shown in Figure 5. Structure C’s ultimate strength was 93.046 Mpa. Structures D and E showed the highest and lowest ultimate strength, which were108.378 Mpa and 53.706 Mpa, respectively. The Young modulus of the scaffold was calculated based on Hooke’s law in the near-linear region (the curve is calculated by strain from 15 to 20% [42,43]). Structures A, B, and D showed similar Young modulus values, which were 256.24 Mpa, 258.81 Mpa, and 252 Mpa, respectively. This is because the load-bearing beams (0.5 mm diameter beams) in structures A and B are strong enough to withstand loadings. Structure D has the same Young modulus because the main load-bearing structure is the 0.8 mm × 0.8 mm solid cuboid beam in the middle. Even though there will be some failure during the loading for the 0.25 mm diameter side beams, the overall deformation of scaffold D in the axial direction mainly depends on the middle beam. Unlike these three structures, the side supporting beams in structure C, with a diameter of 0.2 mm, would have less resistance to deformation than those with high-strength load-bearing beams, not to mention structure E with few load-bearing beams. This is why the Young modulus for structure C and E is 208.93 Mpa and 160.12 Mpa, respectively.

### 3.2. Model Evaluation of the Scaffold Structure

**Figure 6:**
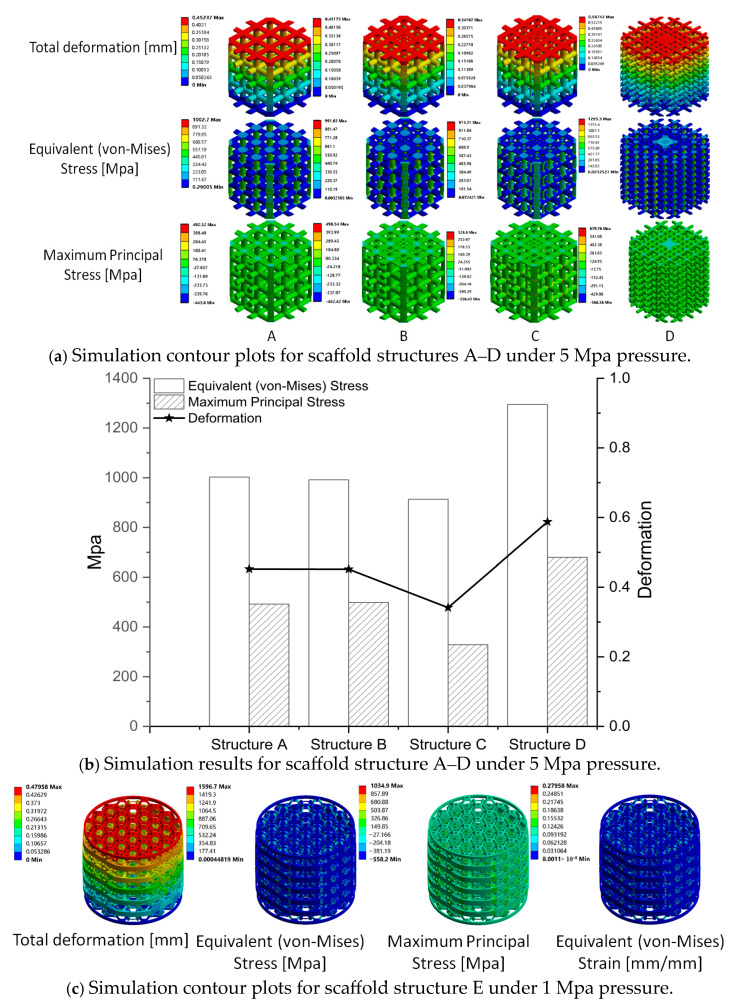
Simulation results for 5 different scaffold structures ((**a**) simulation contour plots for scaffold structure A–D under 5 Mpa pressure; (**b**) simulation results for scaffold structure A–D under 5 Mpa pressure; (**c**) simulation contour plots for scaffold structure E under 1 Mpa pressure).

The simulation models aim to investigate the mechanical performance of the scaffold under steady pressure (5 Mpa for scaffold structures A–D and 1 Mpa for scaffold structure E). Figure 6a displays the contour plot of total deformation, equivalent (von Mises) stress, and maximum principal stress, while Figure 6b presents the maximum values for these result types. Structures A and B demonstrated the same mechanical performance, as the load-bearing beams (0.5 mm diameter) for both structures are the same. Structure C, which is similar to structure B but with more load-bearing beams (0.25 mm diameter), exhibited lower deformation, equivalent (von Mises) stress, and maximum principal stress. Conversely, structure D displayed weaker mechanical performance than structures A, B, and C, compared with the bioreactor results. Figure 6c illustrates the mechanical performance of structure E under 1 Mpa pressure. Given that the failure limit of the material is 1100 Mpa and that structure E’s maximum principal stress reached 1034.9 Mpa under 1 Mpa pressure, it is not recommended for patients with an implanted E scaffold to engage in daily activities.

## 4. Discussion

One of the important factors in implants not having a good bio-performance is the stress shielding problem. A good implant design should balance porosity and mechanical properties. In our design policy, we considered the porosity and stress shielding at first, which characterize structures A–D. Unlike the other structures, scaffold E does not suffer from high stress, but it is still a suitable choice for elderly people. Most elderly OA patients suffer severe pain, and a cane or wheelchair is normally used in their lifetime, which means the scaffold does not need to tolerate high stress compared to those in patients at other OA stages.

According to Kutzner et al., loads on the knee joint during activities are calculated by body weight (BW). Normal activities like stair ascending and descending (316% BW and 346% BW) might be somewhat difficult for patients if the tissues surrounding the scaffold are not strong enough, as is the case with scaffold structures A–D. Other activities such as level walking (261% BW), knee bending (253% BW), standing up (246% BW), and sitting down (225% BW) are all manageable for patients who have implanted scaffolds with structures A–D [44]. Severe OA patients with the structure E implant would just be allowed to engage in level walking with a cane or a wheelchair and could not perform regular activities.

Scaffold structure A, designed as a common cross-linked structure, is suitable for patients with high bone mesenchymal stem cell (BMSC) activity, typically younger patients. Structure B, with similar biomechanical performance to A, is also suitable for the same patient group but offers higher healing performance due to its gradient design based on nutrient concentration distribution. Structure C, with more beams than structure B, is designed for patients with less active BMSCs but high bone strength. Structure D is intended for patients with more severe osteoarthritis (OA) and weaker bone properties. Structure E is specifically for patients with both OA and osteoporosis, who have limited ability to engage in normal activities. Structures A, B, and C are designed for patients with a high ability to recover, while structures D and E are for patients who need to avoid or delay joint replacement surgery.

The developed biomechanical bioreactor simulates the human knee joint and can help researchers investigate the scaffold’s approximate real bioenvironment during loading. However, there are still some limitations that need to be addressed in the future. Generally, a scaffold implanted in the human or animal body would experience variable pressure, and surrounding tissues would share the pressure. As the universal testing machine cannot provide constant pressure, and even if it could provide increasing force like in Kutzner et al. [44], the scaffold deformation would not show significant differences unless the force caused scaffold failure. Therefore, further improvements should be made to this bioreactor to better evaluate the mechanical performance of the scaffold. Moreover, as finite element analysis has identified the dynamic loading conditions [45,46], future simulation works should be carried out.

## 5. Conclusions

In conclusion, we designed five different scaffold structures, manufactured by AM, to cater to different stages of OA and varying ages or bone mechanical properties. Structures A, B, and C are intended for young OA patients or patients with good bone mechanical properties. Structures D and E are designed for patients who need to avoid or delay joint replacement surgery.

Patients who have structures A–D implanted are able to perform activities such as stair ascending and descending, level walking, knee bending, standing up, and sitting down. However, patients with the structure E scaffold are not able to perform the above activities. This structure is specifically designed for elderly patients with OA and osteoporosis.

We developed a biomechanical bioreactor that simulates the human knee joint to evaluate scaffold design and attempted to mimic the real environment of the scaffold when implanted in the human or animal knee joint. The bioreactor demonstrated good evaluation ability, but further improvements are needed in the future.

## Data Availability

The data reported in this study can be obtained by submitting a request to the corresponding author. Due to privacy considerations, these data are not publicly accessible.
